# Broad-scale ecological niches of pathogens vectored by the ticks *Ixodes scapularis* and *Amblyomma americanum* in North America

**DOI:** 10.7717/peerj.17944

**Published:** 2024-08-23

**Authors:** Abdelghafar Alkishe, Marlon E. Cobos, A. Townsend Peterson

**Affiliations:** 1 Department of Ecology and Evolutionary Biology & Biodiversity Institute, University of Kansas, Lawrence, KS, United States of America; 2 Zoology Department/Faculty of Science, University of Tripoli, Tripoli, Libya; 3 Fish and Wildlife Conservation, Virginia Polytechnic Institute and State University (Virginia Tech), Blacksburg, VA, United States of America

**Keywords:** Ticks, Vector-borne disease, Tick-borne pathogens, National ecological observatory network, Multivariate test, Univariate test

## Abstract

Environmental dimensions, such as temperature, precipitation, humidity, and vegetation type, influence the activity, survival, and geographic distribution of tick species. Ticks are vectors of various pathogens that cause disease in humans, and *Ixodes scapularis* and *Amblyomma americanum* are among the tick species that transmit pathogens to humans across the central and eastern United States. Although their potential geographic distributions have been assessed broadly *via* ecological niche modeling, no comprehensive study has compared ecological niche signals between ticks and tick-borne pathogens. We took advantage of National Ecological Observatory Network (NEON) data for these two tick species and associated bacteria pathogens across North America. We used two novel statistical tests that consider sampling and absence data explicitly to perform these explorations: a univariate analysis based on randomization and resampling, and a permutational multivariate analysis of variance. Based on univariate analyses, in *Amblyomma americanum*, three pathogens *(Borrelia lonestari*, *Ehrlichia chaffeensis*, and *E. ewingii*) were tested; pathogens showed nonrandom distribution in at least one environmental dimension. Based on the PERMANOVA test, the null hypothesis that the environmental position and variation of pathogen-positive samples are equivalent to those of *A. americanum* could not be rejected for any of the pathogens, except for the pathogen *E. ewingii* in maximum and minimum vapor pressure and minimum temperature. For *Ixodes scapularis,* six pathogens (*A. phagocytophilum*,* Babesia microti*, *Borrelia burgdorferi* sensu lato, *B. mayonii*, *B. miyamotoi*, and *Ehrlichia muris*-like) were tested; only *B. miyamotoi* was not distinct from null expectations in all environmental dimensions, based on univariate tests. In the PERMANOVA analyses, the pathogens departed from null expectations for *B. microti* and *B. burgdorferi* sensu lato, with smaller niches in *B. microti*, and larger niches in *B. burgdorferi* sensu lato, than the vector. More generally, this study shows the value of large-scale data resources with consistent sampling methods, and known absences of key pathogens in particular samples, for answering public health questions, such as the relationship of presence and absence of pathogens in their hosts respect to environmental conditions.

## Introduction

Ticks are important vectors of various pathogens including *Borrelia burgdorferi* sensu stricto, *Babesia microti*, *B. divergens*, *B. venatorum*, *B. duncani*, *Anaplasma phagocytophilum*, *Ehrlichia chaffeensis*, *Rickettsia rickettsii*, *R. parkeri*, and *Francisella tularensis* (Sonenshine, 2018). *Ixodes scapularis* (black-legged tick) and *Amblyomma americanum* (Lone Star tick) are among the tick species that transmit pathogens to humans across the central and eastern United States (CDC, 2022a; CDC, 2022b; CDC, 2022c). These two tick species have expanded their geographic distributions and established new populations in recent years (Eisen et al., 2016; Molaei et al., 2019; Robinson et al., 2022). Such shifts in tick geographic distributions are expected to influence the epidemiology of pathogens transmitted by these ticks (Parham et al., 2015; Patz et al., 2000).

In the United States, around 476,000 people are known to have contracted Lyme disease per year, such that many more are likely at risk to some degree (CDC, 2022a; CDC, 2022b; CDC, 2022c). The number of reported cases has increased recently (18,000 in 2000 to 42,743 in 2017; CDC, 2022a; CDC, 2022b; CDC, 2022c) following patterns of temperature increase, increasing surveillance effort, landscape change, and host population changes (Alkishe, Raghavan & Peterson, 2021; Diuk-Wasser, Van Acker & Fernandez, 2021; McMahon, Morand & Gray, 2018; Hansen et al., 2001), with most human cases documented in the northeastern United States (*e.g.*, 3-year average incidence 14.1 in District of Columbia and 116.5 in Maine; CDC, 2022a; CDC, 2022b; CDC, 2022c). In Canada, likely owing to change in temperature and precipitation patterns, newly established populations of *I. scapularis* have caused increases in Lyme disease cases in the region, from 144 cases in 2009 to 2,168 cases in 2022 (Wilson et al., 2022; Government of Canada, 2023).

However, some marked contrasts exist between the geographic distributions of vector tick species and those of human cases (CDC, 2022a; CDC, 2022b; CDC, 2022c). For example, Lyme disease cases are concentrated in two major focal areas in the northeastern and northcentral United States, whereas the vector tick (*I. scapularis*) has a much broader distribution in eastern North America (Peterson & Raghavan, 2017), with few or no human cases reported in the southern and western parts of the distribution of the species (CDC, 2022a; CDC, 2022b; CDC, 2022c). The reasons for this contrast have not yet been understood; possibilities include lack of awareness about Lyme disease, absence of the pathogen, or presence of incompetent host populations in those areas (Radolf et al., 2021).

Environmental factors such as temperature, precipitation, humidity, and vegetation type influence the activity, survival, and geographic distribution of different tick species (Ogden et al., 2021; Paul et al., 2016). For example, *I. scapularis* nymphal development does not occur below 0 °C or above 32 °C, with faster development rates at warmer temperatures within this range; in general, though its life cycle length is determined by temperatures (Ogden et al., 2004). *Ixodes scapularis* nymphs are active from March to October, peaking from May to July, in northeastern states (Eisen et al., 2016); their active season is slightly later in the extreme northern United States and southeastern Canada (April to November; Arsnoe, Tsao & Hickling, 2019). For *A. americanum*, nymphs are active from May to September in Georgia, and from May to August in Missouri (Davidson, Siefken & Creekmore, 1994; Kollars Jr et al., 2000). However, nymphs are considered to present greatest risk for pathogen transmission, at least in part owing to their small size compared to adult ticks (Heyman et al., 2010).

Ticks and tick-borne diseases are susceptible to climate change, as increased temperature in areas with cold or temperate environments leads to longer periods of survival and activity for ticks, and can cause diseases to emerge and reemerge (Bouchard et al., 2019; Hroobi et al., 2021; Raghavan et al., 2021). Reservoir hosts play crucial roles in maintaining and spreading pathogens, yet are difficult to consider owing to their diversity and complexity (Gibb et al., 2020; Salomon et al., 2021). Ecological niche modeling techniques have been used extensively to understand and anticipate impacts of changing climates on the geographic distributions of different tick species in North America (Alkishe et al., 2020; Alkishe & Peterson, 2021; Alkishe & Peterson, 2022; Boorgula et al., 2020; Burtis et al., 2022; Raghavan et al., 2019; Ripoche et al., 2022). However, understanding of the relationships among the ecological niches of vectors and associated pathogens in environmental dimensions in North America remains incipient.

In this contribution, we took advantage of the large-scale data resources of the National Ecological Observatory Network (NEON). In particular, we analyzed two tick species (*I. scapularis*, and *A. americanum*) and associated bacterial pathogens. NEON protocols include regular sampling of different tick species and life stages using a dragging method. Tick samples collected by NEON personnel are identified by experts to species, life stage, and sex; nymphs are tested for presence or absence of bacterial and protozoan pathogens (NEON, 2022a). Because no comprehensive study has yet been developed to explore associations between ticks and associated pathogens, we used the NEON data to achieve broad geographic coverage. We also employed new statistical approaches (Cobos & Peterson, 2022) to explore and test signals of ecological niches of pathogens, taking the sampling of ticks into account explicitly to obtain absence data as well as presence data for pathogens. In effect, the question being tested is whether the pathogen species have sets of environmental requirements distinct from those of their tick vectors, or whether they are present where the ticks are present, in terms of environmental conditions.

## Methods

### Ticks and tick-borne pathogen data sets

Data packets summarizing detections of ticks and tick-borne pathogens were downloaded from the National Ecological Observatory Network (NEON, available at https://data.neonscience.org/data-products/DP1.10092.001; NEON, 2022b) for *A. americanum* and *I. scapularis* (2014–2020) with a total of 59 and 39 sites sampled annually for *A. americanum* and *I. scapularis*, respectively. We applied a data cleaning process to remove records holding errors such as missing information or incorrect pathogen names. We ended up with 71,113 and 16,800 individual counts of *A. americanum* and *I. scapularis*, with 12 and 13 pathogens tested, respectively (Table 1). We excluded pathogens with <2,000 test results for lack of statistical power, pathogens that are not considered to be transmitted effectively by the vector (*e.g.*, *A. americanum, A. phagocytophilum,* and *Borrelia burgdorferi* sensu lato), and “*Borrelia* sp” from our analysis given uncertainty regarding the identity of pathogen species. After data cleaning, we explored geographic patterns of prevalence for each tick and pathogen combination.

**Table 1:** Summary of ticks and tick-borne pathogens (cleaned data) from NEON.

Pathogens	Negative	Positive	Total tests	Prevalence
*Amblyomma americanum*				
*Anaplasma phagocytophilum*	10,155	4	10,159	0.039
*Babesia microti*	37	0	37	0
*Borrelia burgdorferi* sensu lato	28	9	37	24.32
*Borrelia lonestari*	10,042	80	10,122	0.79
*Borrelia mayonii*	37	0	37	0
*Borrelia miyamotoi*	36	1	37	2.70
*Borrelia* sp.	10,068	91	10,159	0.89
*Ehrlichia chaffeensis*	10,074	48	10,122	0.47
*Ehrlichia ewingii*	10,075	47	10,122	0.46
*Ehrlichia muris*-like	37	0	37	0
*Francisella tularensis*	10,122	0	10,122	0
*Rickettsia rickettsii*	10,122	0	10,122	0
Grand Total	70,833	280	71,113	0.39
*Ixodes scapularis*				
*Anaplasma phagocytophilum*	2,319	80	2,399	3.33
*Babesia microti*	2,180	63	2,243	2.80
*Borrelia burgdorferi*	7	0	7	0
*Borrelia burgdorferi* sensu lato	1,813	430	2,243	19.17
*Borrelia lonestari*	156	0	156	0
*Borrelia mayonii*	2,230	13	2,243	0.57
*Borrelia miyamotoi*	2,211	32	2,243	1.42
*Borrelia* sp.	1,944	455	2,399	18.96
*Ehrlichia chaffeensis*	156	0	156	0
*Ehrlichia ewingii*	156	0	156	0
*Ehrlichia muris*-like	2,234	9	2,243	0.40
*Francisella tularensis*	156	0	156	0
*Rickettsia rickettsii*	156	0	156	0
Grand Total	15,718	1,082	16,800	6.44

### Environmental data

We downloaded raster-format data layers summarizing monthly averages of maximum temperature, minimum temperature, mean temperature, maximum vapor pressure deficit, and minimum vapor pressure deficit, from the PRISM climate data archive at 4 km spatial resolution (available at https://prism.oregonstate.edu/recent/). Vapor pressure was incorporated in these analyses because it is a better reflection of water availability than precipitation (Cáceres et al., 2007). We selected these variables as they have been considered important in determining nymphal tick abundance (Bertrand & Wilson, 1996; Vail & Smith, 2002). Time periods for the variables downloaded corresponded to the month of sampling and the month previous to sampling. We took the mean of values for these two months, and created four different sets of environmental variables for each record to explore different combinations of environmental information: set 1 (minimum temperature, maximum vapor pressure deficit), set 2 (minimum temperature, minimum vapor pressure deficit), set 3 (maximum and minimum vapor pressure deficit), and set 4 (minimum and maximum vapor pressure deficit, minimum temperature). We ended up using only set 4 after assessing linear correlations, which contains three variables (minimum and maximum vapor pressure deficit, minimum temperature). Set 4 was the only set of environmental variables for which all pairwise correlation coefficients were $ \left\vert r \right\vert < 0.8$. We performed all of these preparatory analyses using the packages raster (Hijmans, 2019) and stats in R version 4.2.2 (R Core Team, 2017).

### Pathogen niche exploration

We created visualizations of environmental conditions used by the two ticks and associated pathogens compared to monthly-specific conditions in the United States using three variables (minimum temperature, maximum vapor pressure deficit, and minimum vapor pressure deficit). We used two statistical tests to detect signals of pathogen ecological niche in data derived from testing ticks for such pathogens, and thus taking into account both presence and absence data in the NEON testing results. First, permutational multivariate analyses of variance (PERMANOVA; Anderson, 2017) were used to detect overall signals of niche difference between tick and pathogen, comparing the complete data set (positive and negative test results) against positive cases for detection of the pathogen.

This analysis allowed us to test the null hypothesis that either the position (centroid) or the spread (dispersion) of the two samples are equivalent (Cobos & Peterson, 2022). This hypothesis is rejected when the position and/or dispersion of the samples are not demonstrably equivalent. Second, non-parametric univariate analyses were used to understand changes in position and breadth of pathogen niches compared to null distributions of those niche characteristics derived from resampling from the whole dataset (Cobos & Peterson, 2022). The null hypothesis in this test is that the pathogen niche position or breadth cannot be distinguished from that of the tick. Mean, median, standard deviation (SD), and range were used to test the null hypothesis. When the hypothesis was rejected, the pathogen niche position (mean and median) or breadth (SD and range) could be lower (*i.e.,* observed value below the 2.5th percentile of the null distribution derived from all data) or higher (*i.e.,* observed value above the 97.5th percentile of the null distribution). We performed these tests using 1,000 random samples of size equivalent to the number of positive tests from the overall dataset to generate null distributions against which the observed value of the statistic was compared. It is worth mentioning that, as the two tests are based on different concepts and procedures, they complement one other, in the sense that they both help to detect signals of pathogen environmental preference; the univariate approach aids in interpretation of the type of signal more explicitly than the multivariate tests. All analyses described above were performed in R, using functions available at https://github.com/marlonecobos/host-pathogen (Cobos & Peterson, 2022); the code used for our examples is available at https://github.com/Abduelkeesh/Broad-scale-ecological-niche-of-pathogens.git.

**Table 2:** Results derived from the univariate niche comparisons for bacterial pathogens detected in the tick *Amblyomma americanum*, with lower or higher representing statistically significant signals for the observed value (more extreme than the central 95%). Variables assessed include minimum temperature (tmin), maximum vapor pressure deficit (vpdmax), and minimum vapor pressure deficit (vpdmin).

	Central tendency		Variation	
Variables	Mean	Median	Standard deviation	Range
*Borrelia lonestari*				
tmin	–	–	–	–
vpdmax	lower	lower	higher	–
vpdmin	–	–	–	higher
*Ehrlichia chaffeensis*				
tmin	–	–	–	higher
vpdmax	–	higher	higher	–
vpdmin	–	–	higher	–
*Ehrlichia ewingii*			
tmin	lower	–		–
vpdmax	–	–	higher	higher
vpdmin	–	higher	lower	

## Results

After data cleaning, we had data on occurrence and pathogen status for 71,113 *A. americanum* ticks for 12 pathogens, and for 16,800 *I. scapularis* ticks for 13 pathogens. After removing from consideration pathogens with small sample sizes and those with zero prevalences, we had 40,525 (57% of the data), and 13,614 (81% of the data) ticks tested for three and six pathogens for *A. americanum* and *I. scapularis*, respectively (Tables 1–3). *Borrelia lonestari*, *Ehrlichia chaffeensis*, and *E. ewingii* were therefore explored for *Amblyomma americanum*. *Anaplasma phagocytophilum*, *Babesia microti*, *Borrelia burgdorferi* sensu lato, *B. mayonii*, *B. miyamotoi*, and *Ehrlichia muris*-like, were explored for *I. scapularis* records.

**Figure 1:**
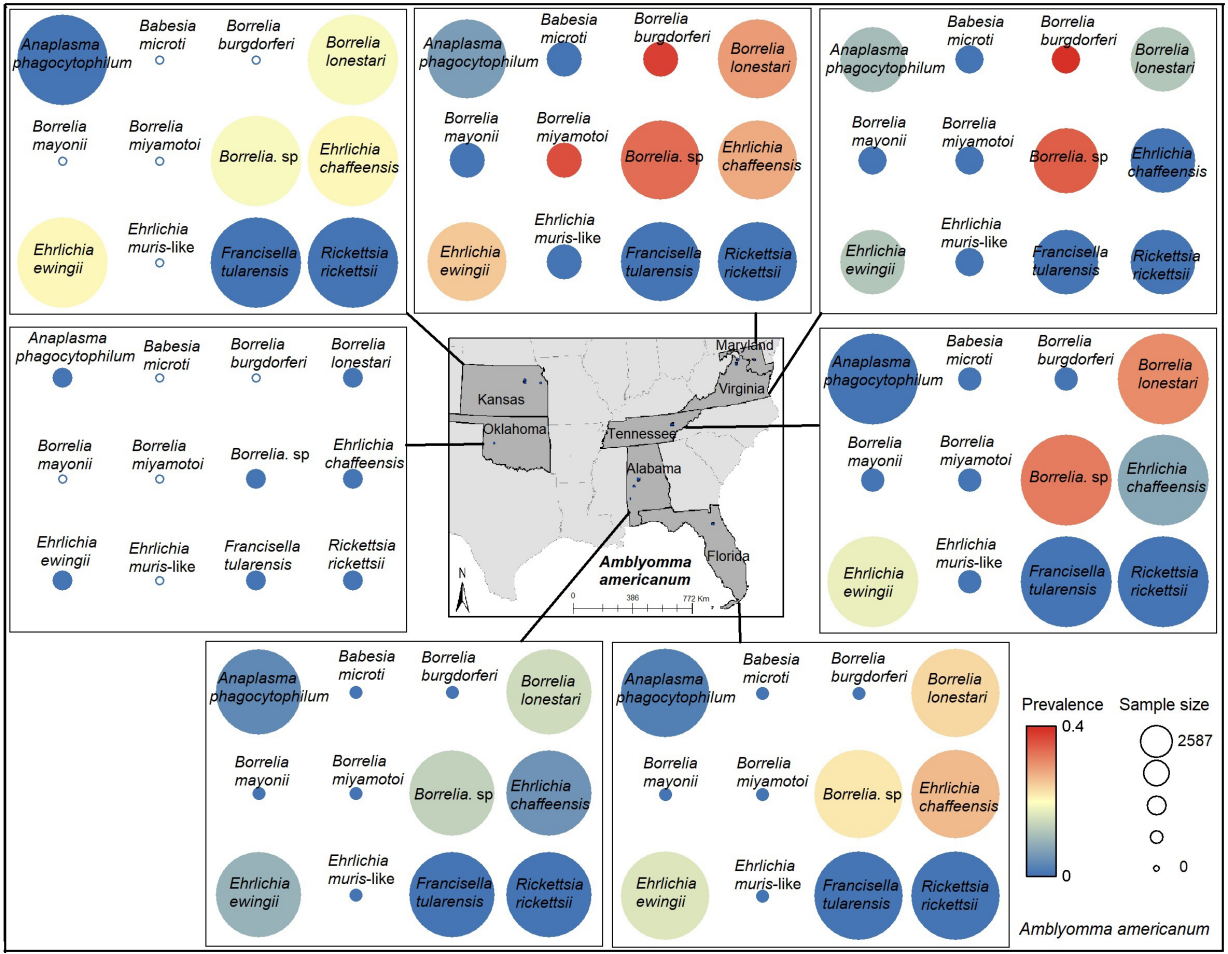
Sample size and prevalence of pathogens in *Amblyomma americanum* from collection sites across seven states. Map data source credit: GADM (https://gadm.org).

Sample sizes differed between the two tick species (Table 1) owing to geographic differences in their range limits and abundances across eastern North America. The two species of ticks were collected at NEON sites during 2014–2020 in several locations across the central and eastern United States (Fig. 1). *Amblyomma americanum* was collected from seven states: Kansas (15 locations), Oklahoma (one location), Alabama (15 locations), Tennessee (six locations), Florida (seven locations), Virginia (nine locations), and Maryland (six locations). *Ixodes scapularis* was sampled in six states: Wisconsin (seven locations), Massachusetts (seven locations), Maryland (six locations), Virginia (12 locations), Tennessee (six locations), and Alabama (one location). Prevalences of pathogens in *A. americanum*ranged 0.1%−1.2% among sites and years; prevalences of pathogens in *I. scapularis* ranged 2.5%-15.6% among sites and years.

**Table 3:** Results derived from the univariate niche comparisons for bacterial pathogens detected in the tick *Ixodes scapularis*, with lower or higher representing statistically significant signals for the observed value (more extreme than the central 95%). Variables assessed include minimum temperature (tmin), maximum vapor pressure deficit (vpdmax), and minimum vapor pressure deficit (vpdmin).

	Central tendency		Variation	
Variables	Mean	Median	Standard deviation	Range
*Anaplasma phagocytophilum*				
tmin	–	–	–	–
vpdmax	–	–	–	lower
vpdmin	–	–	–	lower
*Babesia microti*				
tmin	lower	–	lower	lower
vpdmax	lower	–	lower	lower
vpdmin	lower	–	lower	lower
*Borrelia burgdorferi* sensu lato				
tmin	lower	–	lower	higher
vpdmax	lower	–	lower	–
vpdmin	–	–	lower	higher
*Borrelia mayonii*				
tmin	lower	lower	lower	lower
vpdmax	lower	–	lower	lower
vpdmin	lower	–	lower	lower
*Babesia miyamotoi*	
tmin	–	–	–	–
vpdmax	–	–	–	–
vpdmin	–	–	–	–
*Ehrlichia muris*-like	
tmin	–	–	lower	lower
vpdmax	–	–	lower	lower
vpdmin	–	–	lower	lower

**Figure 2:**
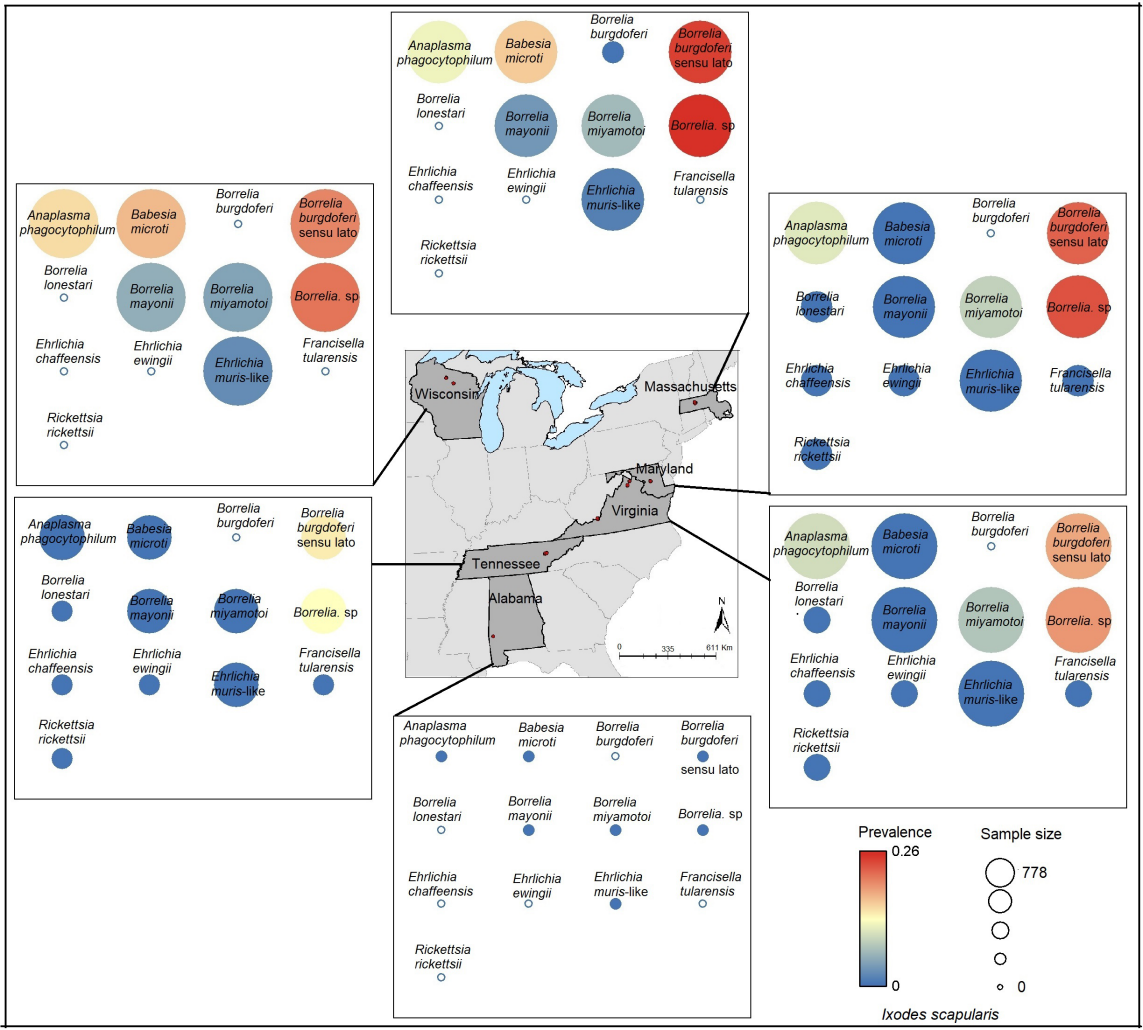
Sample size and prevalence of pathogens in *Ixodes scapularis* from collection sites across six states. Map data source credit: GADM (https://gadm.org).

Considering sample sizes, prevalence of *A. americanum*-associated pathogens was high for *B. lonestari*, in Kansas, Maryland, Tennessee, and Florida, compared with other states (Fig. 1). Even though sample size was low for *B. burgdorferi* sensu lato and *B. miyamotoi*, prevalence was high for those two pathogens in Maryland; in Virginia, prevalence was high only for *B. burgdorferi* sensu lato (Fig. 1). The prevalence of *I. scapularis*-associated pathogens was high for *B. burgdorferi* sensu lato Massachusetts, Wisconsin, Maryland, and Virginia; in Tennessee, prevalence was notably lower (Fig. 2).

### *Amblyomma americanum* and its pathogens

**Figure 3:**
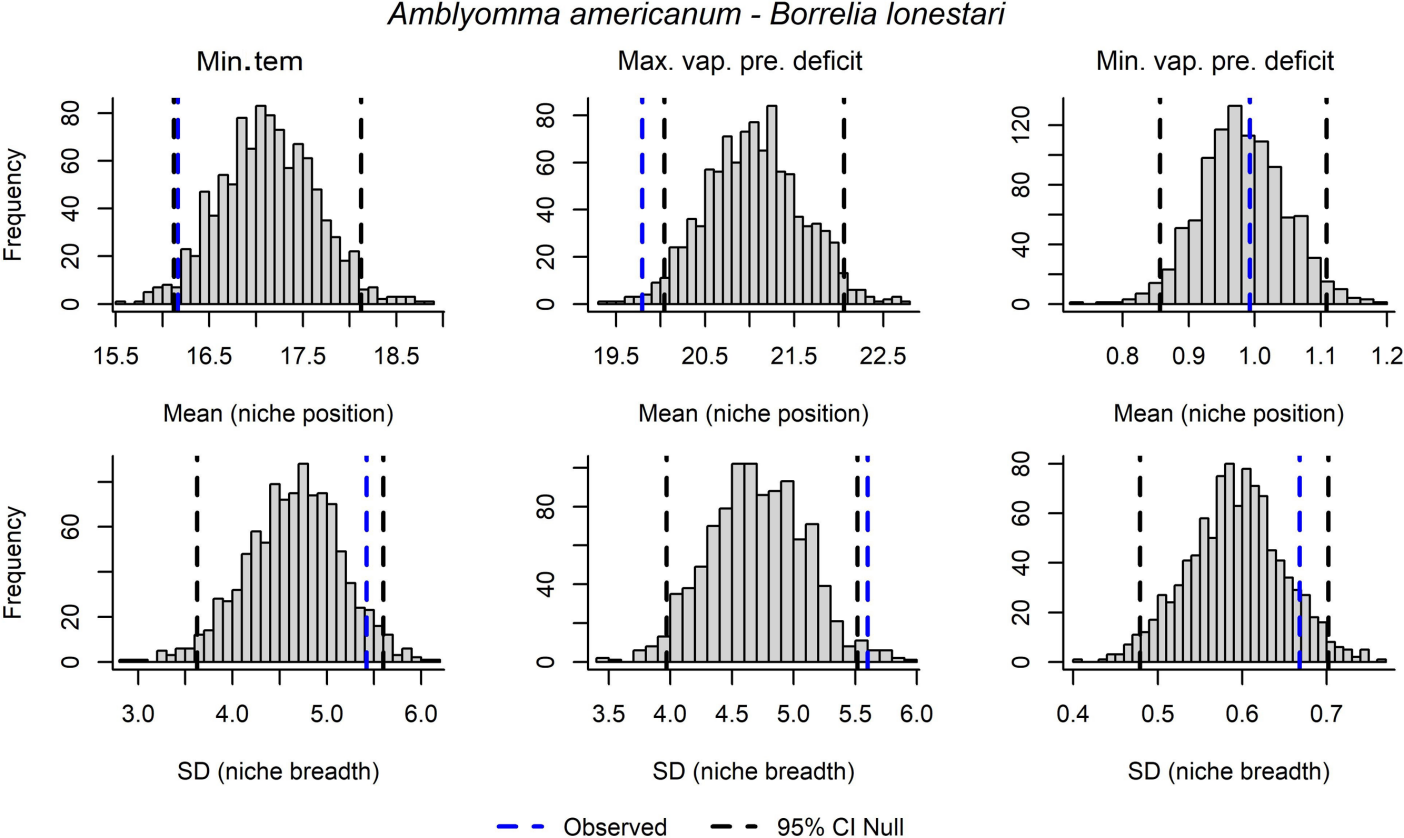
Results from univariate non-parametric tests to detect signals of niche for the pathogen *Borrelia lonestari* in *Amblyomma americanum*. Variables assessed include minimum temperature (Min.tem), maximum vapor pressure deficit (Max.vap.pre.deficit), and minimum vapor pressure deficit (Min.vap.pre.deficit).

Results from univariate tests showed significant signals of pathogen niche for *B. lonestari* in *A. americanum* in niche position and breadth, for maximum vapor pressure deficit (Table 2, Fig. 3). For *E. chaffeensis*, a significant signal of niche was detected in niche breadth and for minimum temperature (Table 2). A significant pathogen niche signal was detected for *E. ewingii* for niche position in terms of minimum temperature, and for niche breadth in maximum vapor pressure. For this pathogen, minimum vapor pressure showed significant signals in both niche breadth and position (Table 2; Table S1).

**Figure 4:**
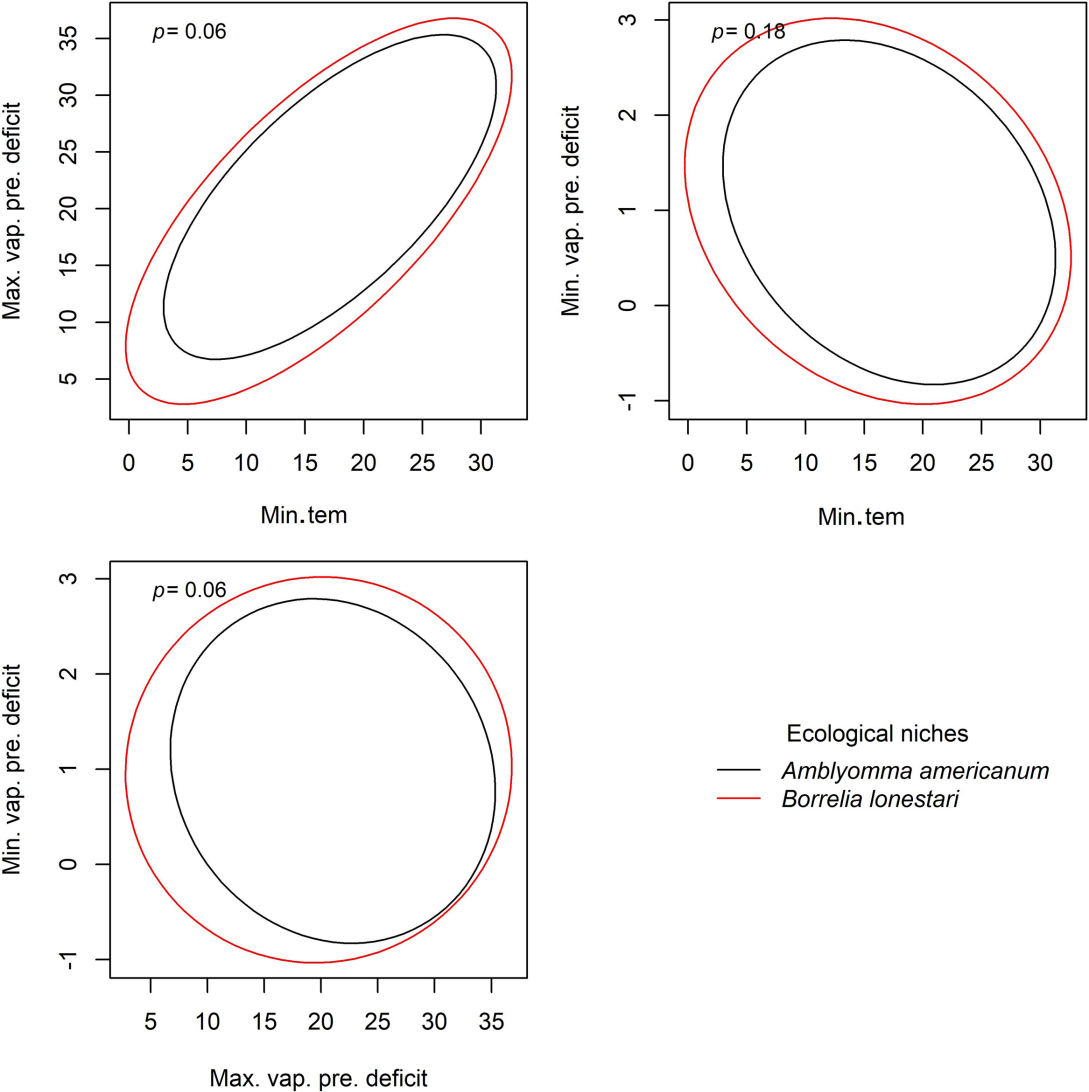
Results from PERMANOVA analyses to detect signals of ecological niche for *Borrelia lonestari* in *Amblyomma americanum*. Ellipses were created with a 95% confidence limit, *P* values are shown for each comparison. Variables assessed include minimum temperature (Min.tem), maximum vapor pressure deficit (Max.vap.pre.deficit), and minimum vapor pressure deficit (Min.vap.pre.deficit).

PERMANOVA results showed that no signal of pathogen ecological niche could be detected for *B. lonestari* in *A. americanum* under two environmental dimensions of maximum vapor pressure deficit and minimum temperature; minimum vapor pressure deficit and minimum temperature; and minimum vapor pressure deficit and maximum vapor pressure deficit (Fig. 4). For *E. ewingii* in *A. americanum*, a signal of niche was detected with PERMANOVA analyses (*p* < 0.05) in two of the explorations performed (Fig. 4, and Fig. S3).

**Figure 5:**
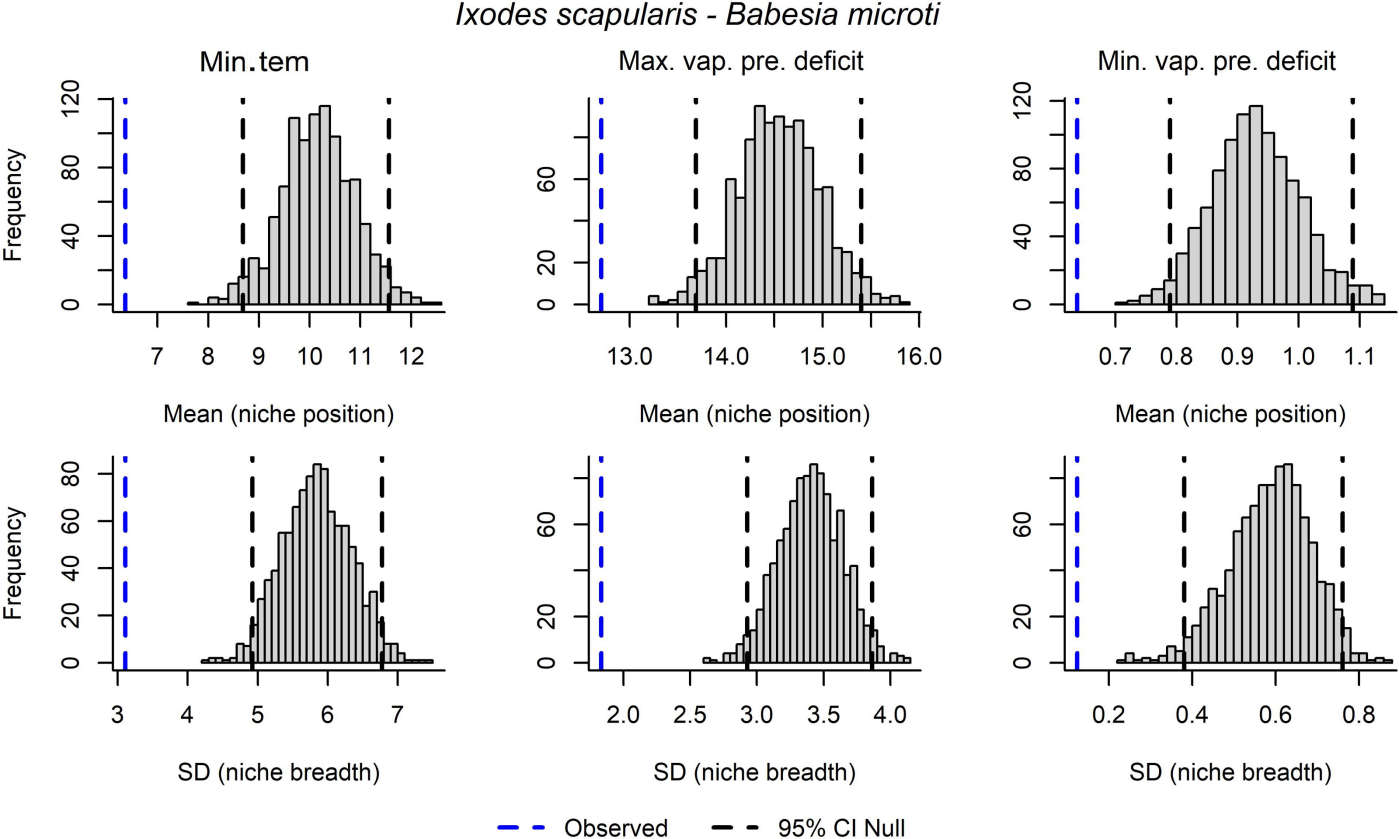
Results from univariate non-parametric tests to detect signals of niche for the pathogen *Babesia microti* in *Ixodes scapularis*. Variables assessed include minimum temperature (Min.tem), maximum vapor pressure deficit (Max.vap.pre.deficit), and minimum vapor pressure deficit (Min.vap.pre.deficit).

**Figure 6:**
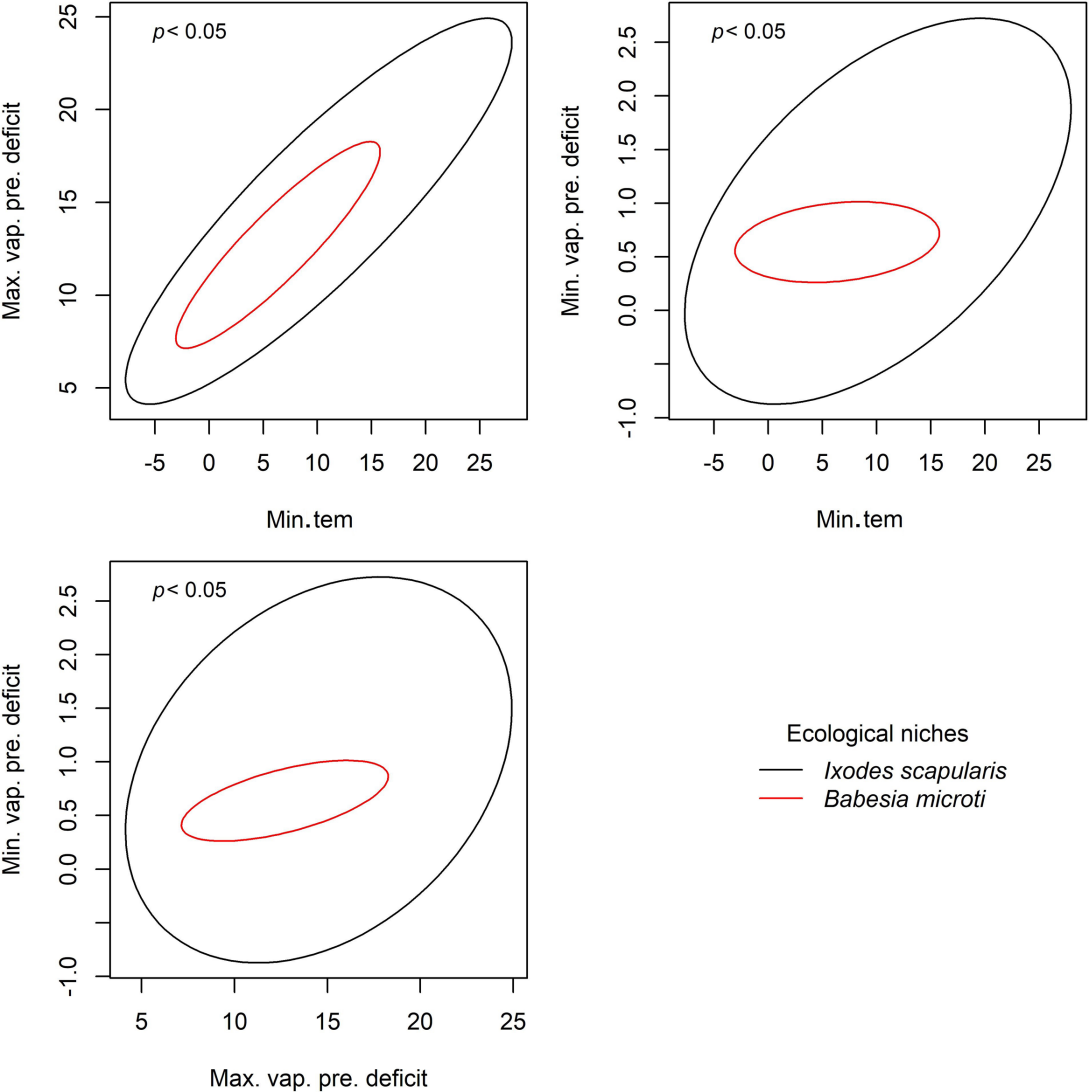
Results from PERMANOVA analyses to detect signals of ecological niche for *Babesia microti* in *Ixodes scapularis*. Ellipses were created with 95% confidence limit, *P* values are shown for each comparison. Variables assessed include minimum temperature (Min.tem), maximum vapor pressure deficit (Max.vap.pre.deficit), and minimum vapor pressure deficit (Min.vap.pre.deficit).

### *Ixodes scapularis* and its pathogens

In univariate analyses, significant signals of pathogen niche were detected in terms of niche breadth for maximum and minimum vapor pressure deficit in *A. phagocytophilum*. For *B. microti*, significant signals of both niche position and breadth were observed for minimum temperature, and maximum and minimum vapor pressure deficit (Fig. 5). For *B. burgdorferi* sensu lato, significant signal for niche position was detected for minimum temperature and maximum vapor pressure deficit; a significant signal for niche breadth was detected in minimum temperature and minimum vapor pressure deficit (Fig. S2). *Borrelia mayonii* showed a significant signal in niche position and breadth for minimum temperature, maximum vapor pressure deficit, and minimum vapor pressure deficit. For *E. muris*-like, a significant signal in terms of niche breadth was found for minimum temperature, maximum vapor pressure deficit, and minimum vapor pressure deficit. For *B. miyamotoi*, no statistically significant results were found (Table 3).

We found statistically significant signals of ecological niche for the pathogens *B. microti* and *B. burgdorferi* sensu lato in *I. scapularis* based on PERMANOVA analyses (Fig. 6 and Fig. S4). The multivariate approach did not detect statistically significant results for any of the other four pathogens explored.

## Discussion

Animal hosts play crucial roles in spreading and maintaining zoonotic pathogens, yet understanding this mechanism is not easy in view of the complex transmission system of many of the pathogens. In tick-borne disease systems, many tick species use a wide variety of wildlife hosts during different life stages, creating complex transmission pathways and opportunities for diverse infections and co-infections. The complexity also comes from other ecological factors that may influence the roles of hosts, ticks, and tick-borne pathogen dynamics, such as the relationship between abundances of important hosts and tick abundances, land-cover pattern and configuration, climate and climate change, and land use (Tsao et al., 2021). In this regard, we focused on understanding the relationship between ticks and their associated pathogens from an environmental perspective using variables describing aspects of climate.

The analyses presented above took advantage of the national-scale tick collections and pathogen testing conducted under the aegis of the National Ecological Observatory Network (NEON). Although NEON has been in operation only since 2006 (NEON, 2022), its broad geographic scope and consistent methodologies implemented across a network of 81 sites (although only 46 have contributed data on ticks) make it a unique resource that makes possible broad-scope, geographic-scale analyses such as those that we have presented. To the extent that NEON achieves long-term sustainability, consistency, quality, and continuity in its data streams, this data resource can illuminate many important facets of biology across the United States.

This study is based on tick collections from locations in seven states for *A. americanum* and six states for *I. scapularis*, with all data drawn from the NEON data repository. These sampling sites were distributed across Kansas, Oklahoma, Tennessee, Alabama, Florida, Virginia, Maryland, Wisconsin, and Massachusetts (Figs. 1 and 2), which may have limited our ability to detect full niche signals, and may produce some bias of importance in interpreting our results in addition to not considering reservoir host biology. Prevalence of *B. burgdorferi* sensu lato (the causative agent of Lyme disease) was high only in *I. scapularis* from the northeastern (Massachusetts, Maryland) and midwestern (Wisconsin) states, where Lyme disease is common (CDC, 2021), but sampling in the NEON program did not cover much of the environmental diversity of sites where that pathogen species occurs. Other caveats that may affect our outcomes include the coverage of environmental diversity represented in the input datasets (*i.e.,* we can only detect differences between niches if they are manifested over the region that was sampled), and sample size effects that magnify statistical power in well-sampled species compared to other species with smaller samples.

We used monthly weather summaries from the PRISM data archive, which allowed us to relate occurrence records to specific environmental conditions corresponding to a particular time and place. This sort of environmental data is coarse-resolution (macroclimate) rather than fine-resolution (microclimate), although the latter may have more direct influences on ticks (Bacon et al., 2022). In this sense, our models may be more about broad geographic range limits, as opposed to the details of abundances and local population fitness (Martínez-Meyer et al., 2013).

However, our goal in this study was to detect niche signals that might contrast between two tick vectors and their many associated bacterial pathogens. Our analyses and graphical explorations allowed us to assess whether pathogen niches exist that may be more than just that the pathogen is following the same tick niches. For instance, *A. americanum* and *B. lonestari* appeared to have similar niche breadths in terms of minimum temperature and minimum vapor pressure deficit (Fig. 3). We saw contrasting niches for *I. scapularis* and its pathogen *B. microti* (Fig. 4), suggesting that pathogens may have environmental requirements that do not always match exactly the environmental requirements of their tick host.

Our analysis appears to be the first in terms of comparing and contrasting ecological niche signals between ticks and tick-borne pathogens in such detail and across such broad scales. MacDonald, McComb & Sambado (2022) assessed local-scale community ecology of Lyme disease and human incidence in California. This study is different, in that we took advantage of the broad-scale sampling and intensive testing of ticks in the NEON initiative, and also a new methodological protocol that controls explicitly for the sampling that produced the set of positive detections in the study (Cobos & Peterson, 2022)—this approach allowed us to test for niche differences while controlling explicitly for sampling intensity. Although our two niche-focused analyses (multivariate and univariate analysis) have different underlying concepts, they complement each other to provide a good tool for detecting signals of niche similarity and dissimilarity (Cobos & Peterson, 2022).

We could not detect any clear signal of dissimilar niches for *A. americanum* and the pathogen *B. lonestari* in any of our analyses except under mean (niche position) of maximum vapor pressure deficit, and SD (niche breadth), where the pathogen has a higher and narrower niche than the tick vector, respectively, based on univariate tests (Fig. 4). We note, however, that univariate and multivariate test results often contrast, given that the former do not consider variable interactions, and that the latter consider the effects of all of the independent variables together.

In this example, the data available for analysis contained few infected ticks compared to the number of samples; most importantly, the somewhat limited number of locations that contributed relevant ticks in the NEON project makes it difficult to detect full signals of niche dissimilarity. It is crucial that we understand the relationship between ticks and tick-borne pathogens (Estrada-Peña et al., 2021). Thus, it is necessary to have data that cover as many areas as possible to detect complete signals of niches and to contrast suitable and unsuitable conditions fully (Soberón & Peterson, 2005).

Finally, the types of analyses performed here can be important in public health mitigation efforts, as they help to understand why a pathogen is in some areas, but not in others, even though the vector tick species occurs similarly. For instance, our findings showed that *B. burgdorferi* does not prefer more arid conditions, which may begin to answer why Lyme disease is not as common in southern and midwestern states, where climate conditions are warmer and less humid than areas to the north and northeast. Such broad-scale assessments become possible only with the combination of broad-scale data availability and the development of specific analytical tools that are appropriate for such questions.

##  Supplemental Information

10.7717/peerj.17944/supp-1 Supplemental Information 1 NEON data set

10.7717/peerj.17944/supp-2 Supplemental Information 2 Supplementary materials

## Additional Information and Declarations

### Data Availability

The following information was supplied regarding data availability: The data packets summarizing detections of ticks and tick-borne pathogens are available at the National Ecological Observatory Network (NEON): https://data.neonscience.org/data-products/DP1.10092.001.

## References

[ref-1] Alkishe A, Cobos ME, Peterson AT, Samy AM (2020). Recognizing sources of uncertainty in disease vector ecological niche models: an example with the tick *Rhipicephalus sanguineus* sensu lato. Perspectives in Ecology and Conservation.

[ref-2] Alkishe A, Peterson AT (2021). Potential geographic distribution of *Ixodes cookei*, the vector of Powassan virus. Journal of Vector Ecology.

[ref-3] Alkishe A, Peterson AT (2022). Climate change influences on the geographic distributional potential of the spotted fever vectors *Amblyomma maculatum* and *Dermacentor andersoni*. PeerJ.

[ref-4] Alkishe A, Raghavan RK, Peterson AT (2021). Likely geographic distributional shifts among medically important tick species and tick-associated diseases under climate change in North America: a review. Insects.

[ref-5] Anderson MJ (2017).

[ref-6] Arsnoe I, Tsao JI, Hickling GJ (2019). Nymphal *Ixodes scapularis* questing behavior explains geographic variation in Lyme borreliosis risk in the eastern United States. Ticks and Tick-Borne Diseases.

[ref-7] Bacon E, Kopsco H, Gronemeyer P, Mateus-Pinilla N, Smith R (2022). Effects of climate on the variation in abundance of three tick species in Illinois. Journal of Medical Entomology.

[ref-8] Bertrand MR, Wilson ML (1996). Microclimate-dependent survival of unfed adult Ixodes scapularis (Acari: Ixodidae) in nature: Life cycle and study design implications. Journal of Medical Entomology.

[ref-9] Boorgula GD, Peterson AT, Foley DH, Ganta RR, Raghavan RK (2020). Assessing the current and future potential geographic distribution of the American dog tick, Dermacentor variabilis (Say) (Acari: Ixodidae) in North America. PLOS ONE.

[ref-10] Bouchard C, Dibernardo A, Koffi J, Wood H, Leighton P, Lindsay L (2019). Climate change and infectious diseases: the challenges: increased risk of tick-borne diseases with climate and environmental changes. Canada Communicable Disease Report.

[ref-11] Burtis JC, Foster E, Schwartz AM, Kugeler KJ, Maes SE, Fleshman AC, Eisen RJ (2022). Predicting distributions of blacklegged ticks (*Ixodes scapularis*), Lyme disease spirochetes (*Borrelia burgdorferi* sensu stricto) and human Lyme disease cases in the eastern United States. Ticks and Tick-borne Diseases.

[ref-12] Cáceres L, Gómez-Silva B, Garró X, Rodríguez V, Monardes V, McKay CP (2007). Relative humidity patterns and fog water precipitation in the Atacama Desert and biological implications. Journal of Geophysical Research: Biogeosciences.

[ref-13] CDC (2021). Lyme disease. https://www.cdc.gov/lyme/data-research/facts-stats/lyme-disease-case-map.html.

[ref-14] CDC (2022a). Disease transmitted by ticks. https://www.cdc.gov/ticks/diseases/index.html.

[ref-15] CDC (2022b). Lyme disease. https://www.cdc.gov/lyme/data-research/facts-stats/lyme-disease-case-map.html.

[ref-16] CDC (2022c). Lyme disease. https://www.cdc.gov/lyme/data-research/facts-stats/?CDC_AAref_Val=https://www.cdc.gov/lyme/datasurveillance/surveillance-data.html.

[ref-17] Cobos ME, Peterson AT (2022). Detecting signals of species’ ecological niches in results of studies with defined sampling protocols: example application to pathogen niches. Biodiversity Informatics.

[ref-18] Davidson WR, Siefken DA, Creekmore LH (1994). Seasonal and annual abundance of *Amblyomma americanum* (Acari: Ixodidae) in central Georgia. Journal of Medical Entomology.

[ref-19] Diuk-Wasser MA, Van Acker MC, Fernandez MP (2021). Impact of land use changes and habitat fragmentation on the eco-epidemiology of tick-borne diseases. Journal of Medical Entomology.

[ref-20] Eisen RJ, Eisen L, Ogden NH, Beard CB (2016). Linkages of weather and climate with *Ixodes scapularis* and *Ixodes pacificus* (Acari: Ixodidae), enzootic transmission of *Borrelia burgdorferi*, and Lyme disease in North America. Journal of Medical Entomology.

[ref-21] Estrada-Peña A, Cevidanes A, Sprong H, Millán J (2021). Pitfalls in tick and tick-borne pathogens research, some recommendations and a call for data sharing. Pathogens.

[ref-22] Gibb R, Redding DW, Chin KQ, Donnelly CA, Blackburn TM, Newbold T, Jones KE (2020). Zoonotic host diversity increases in human-dominated ecosystems. Nature.

[ref-23] Government of Canada (2023). Lyme disease: surveillance. https://www.canada.ca/en/public-health/services/diseases/lyme-disease/surveillance-lyme-disease.html.

[ref-24] Hansen AJ, Neilson RP, Dale VH, Flather CH, Iverson LR, Currie DJ, Shafer S, Cook R, Bartlein PJ (2001). Global change in forests: responses of species, communities, and biomes: interactions between climate change and land use are projected to cause large shifts in biodiversity. BioScience.

[ref-25] Heyman P, Cochez C, Hofhuis A, Van Der Giessen J, Sprong H, Porter SR, Losson B, Saegerman C, Donoso-Mantke O, Niedrig M, Papa A (2010). A clear and present danger: tick-borne diseases in Europe. Expert Review of Anti-Infective Therapy.

[ref-26] Hijmans RJ (2019). Raster. Geographic data analysis and modeling. https://CRAN.R-project.org/package=raster.

[ref-27] Hroobi A, Boorgula GD, Gordon D, Bai J, Goodin D, Anderson G, Wilson S, Staggs A, Raghavan RK (2021). Diversity and seasonality of host-seeking ticks in a peri urban environment in the Central Midwest (USA). PLOS ONE.

[ref-28] Kollars Jr TM, Oliver Jr JH, Durden LA, Kollars PG (2000). Host associations and seasonal activity of *Amblyomma americanum* (Acari: Ixodidae) in Missouri. Journal of Parasitology.

[ref-29] MacDonald AJ, McComb S, Sambado S (2022). Linking Lyme disease ecology and epidemiology: reservoir host identity, not richness, determines tick infection and human disease in California. Environmental Research Letters, 9.

[ref-30] Martínez-Meyer E, Díaz-Porras D, Peterson AT, Yáñez Arenas CY (2013). Ecological niche structure and rangewide abundance patterns of species. Biology Letters.

[ref-31] McMahon BJ, Morand S, Gray JS (2018). Ecosystem change and zoonoses in the anthropocene. Zoonoses Public Health.

[ref-32] Molaei G, Little EA, Williams SC, Stafford KC (2019). Bracing for the worst—range expansion of the Lone Star tick in the northeastern United States. New England Journal of Medicine.

[ref-33] NEON (2022). History. https://www.neonscience.org/about/overview/history.

[ref-34] NEON (2022a). Tick pathogen status. https://data.neonscience.org/data-products/DP1.10092.001.

[ref-35] NEON (2022b). Tick pathogen status. https://data.neonscience.org/.

[ref-36] Ogden NH, Ben Beard C, Ginsberg HS, Tsao JI (2021). Possible effects of climate change on ixodid ticks and the pathogens they transmit: predictions and observations. Journal of Medical Entomology.

[ref-37] Ogden N, Lindsay L, Beauchamp G, Charron D, Maarouf A, O’callaghan C, Waltner-Toews D, Barker I (2004). Investigation of relationships between temperature and developmental rates of tick *Ixodes scapularis* (Acari: Ixodidae) in the laboratory and field. Journal of Medical Entomology.

[ref-38] Parham PE, Waldock J, Christophides GK, Hemming D, Agusto F, Evans KJ, Fefferman N, Gaff H, Gumel A, LaDeau S (2015). Climate, environmental and socio-economic change: weighing up the balance in vector-borne disease transmission. Philosophical Transactions of the Royal Society B: Biological Sciences.

[ref-39] Patz JA, Graczyk TK, Geller N, Vittor AY (2000). Effects of environmental change on emerging parasitic diseases. International Journal for Parasitology.

[ref-40] Paul RE, Cote M, Le Naour E, Bonnet SI (2016). Environmental factors influencing tick densities over seven years in a French suburban forest. Parasites & Vectors.

[ref-41] Peterson AT, Raghavan R (2017). The leading edge of the geographic distribution of Ixodes scapularis (Acari: Ixodidae). Journal of Medical Entomology.

[ref-42] R Core Team (2017). https://www.R-project.org/.

[ref-43] Radolf JD, Strle K, Lemieux JE, Strle F (2021). Lyme disease in humans. Current Issues in Molecular Biology.

[ref-44] Raghavan RK, Koestel ZL, Boorgula G, Hroobi A, Ganta R, Harrington Jr J, Goodin D, Stich RW, Anderson G (2021). Unexpected winter questing activity of ticks in the central Midwestern United States. PLOS ONE.

[ref-45] Raghavan RK, Peterson AT, Cobos ME, Ganta R, Foley D (2019). Current and future distribution of the Lone Star tick, Amblyomma americanum (L.) (Acari: Ixodidae) in North America. PLOS ONE.

[ref-46] Ripoche M, Bouchard C, Irace-Cima A, Leighton P, Thivierge K (2022). Current and future distribution of *Ixodes scapularis* ticks in Québec: field validation of a predictive model. PLOS ONE.

[ref-47] Robinson EL, Jardine CM, Koffi JK, Russell C, Lindsay LR, Dibernardo A, Clow KM (2022). Range expansion of *Ixodes scapularis* and *Borrelia burgdorferi* in Ontario, Canada, from 2017 to 2019. Vector-Borne and Zoonotic Diseases.

[ref-48] Salomon J, Lawrence A, Crews A, Sambado S, Swei A (2021). Host infection and community composition predict vector burden. Oecologia.

[ref-49] Soberón J, Peterson AT (2005). Interpretation of models of fundamental ecological niches and species’ distributional areas. Biodiversity Informatics.

[ref-50] Sonenshine DE (2018). Range expansion of tick disease vectors in North America: implications for spread of tick-borne disease. International Journal of Environmental Research and Public Health.

[ref-51] Tsao JI, Hamer SA, Han S, Sidge JL, Hickling GJ (2021). The contribution of wildlife hosts to the rise of ticks and tick-borne diseases in North America. Journal of Medical Entomology.

[ref-52] Vail SG, Smith G (2002). Vertical movement of posture of blacklegged tick (Acari: Ixodidae) nymphs as a function of temperature and relative humidity in laboratory experiments. Journal of Medical Entomology.

[ref-53] Wilson CH, Gasmi S, Bourgeois A-C, Badcock J, Chahil N, Kulkarni MA, Lee M-K, Lindsay LR, Leighton PA, Morshed MG, Smolarchuk C, Koffi JK (2022). Surveillance for *Ixodes scapularis* and *Ixodes pacificus* ticks and their associated pathogens in Canada, 2019. Canada Communicable Disease Report.

