# Supplementary material for: Broad-scale ecological niches of pathogens vectored by the ticks Ixodes scapularis and Amblyomma americanum in North America
Source: PeerJ. 2024 Aug 23;12:e17944. doi: 10.7717/peerj.17944 (PMC11348911; doi:10.7717/peerj.17944)
Supplement: Supplemental Information 2 [file peerj-12-17944-s002.pdf]

Supplementary materials

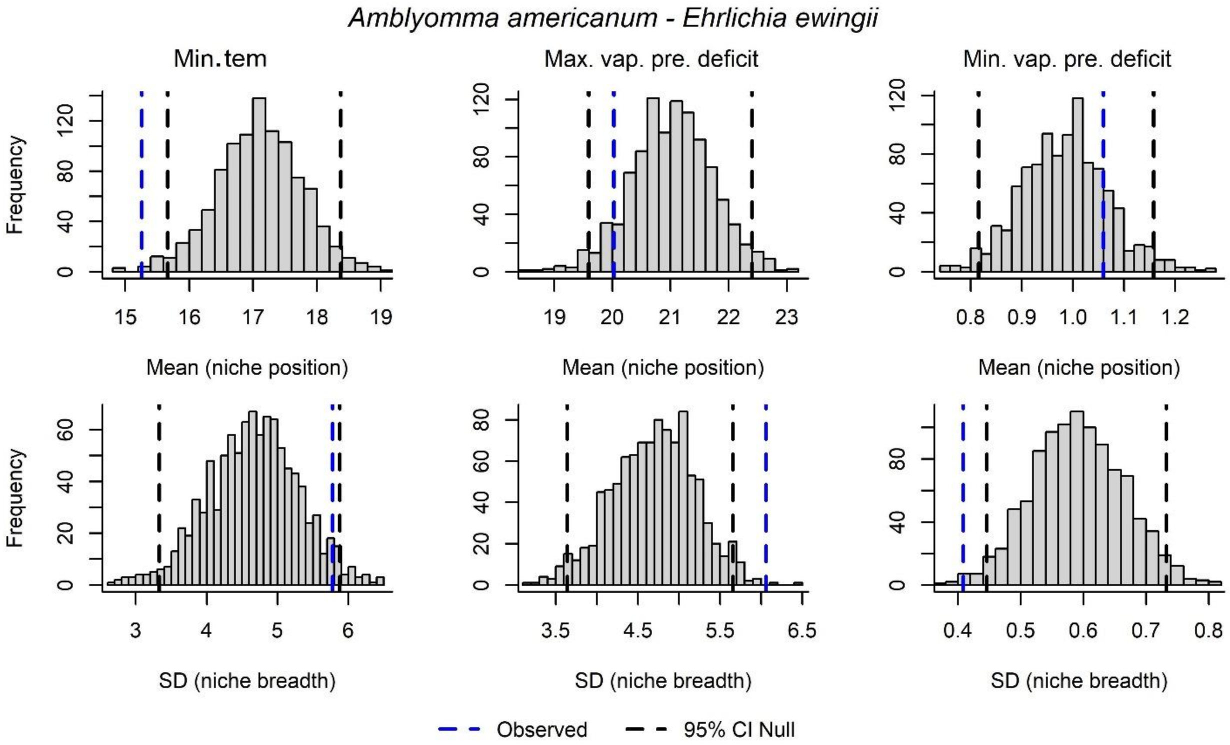

S1. Univariate non-parametric test to detect signals of niche dissimilarity for the pathogen *Ehrlichia ewingii*, and tick species *Amblyomma americanum*. Here we used mean (lower than null maximum vapor pressure deficit) and SD (higher than null maximum vapor pressure deficit) as an example of rejecting the null hypothesis. The null hypnosis was rejected under mean of the minimum temperature, and SD of the maximum and minimum vapor pressure deficit.

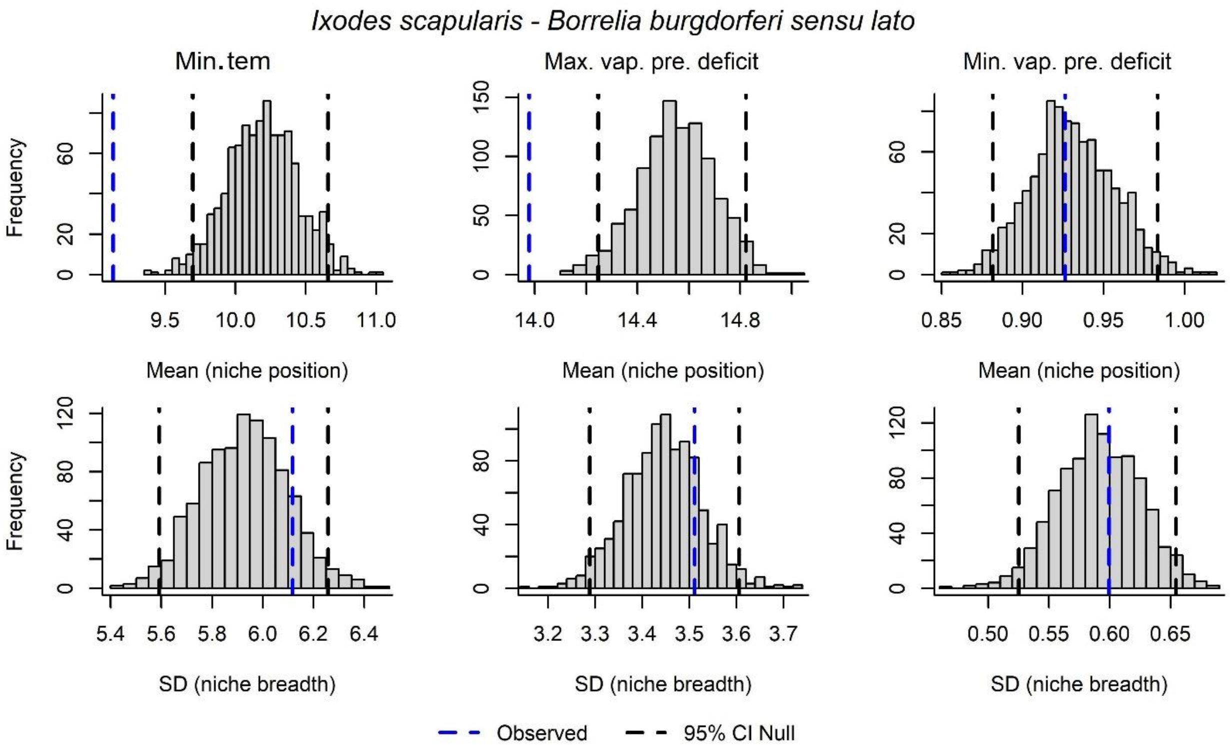

S2. Univariate non-parametric test to detect signals of niche dissimilarity for the pathogen *Borrelia burgdorferi* sensu lato, and tick species *Ixodes scapularis*. Here we used mean (lower than null maximum vapor pressure deficit) and SD (higher than null maximum vapor pressure deficit) as an example of rejecting the null hypothesis. The null hypnosis was rejected under all mean and SD of the environmental variables.

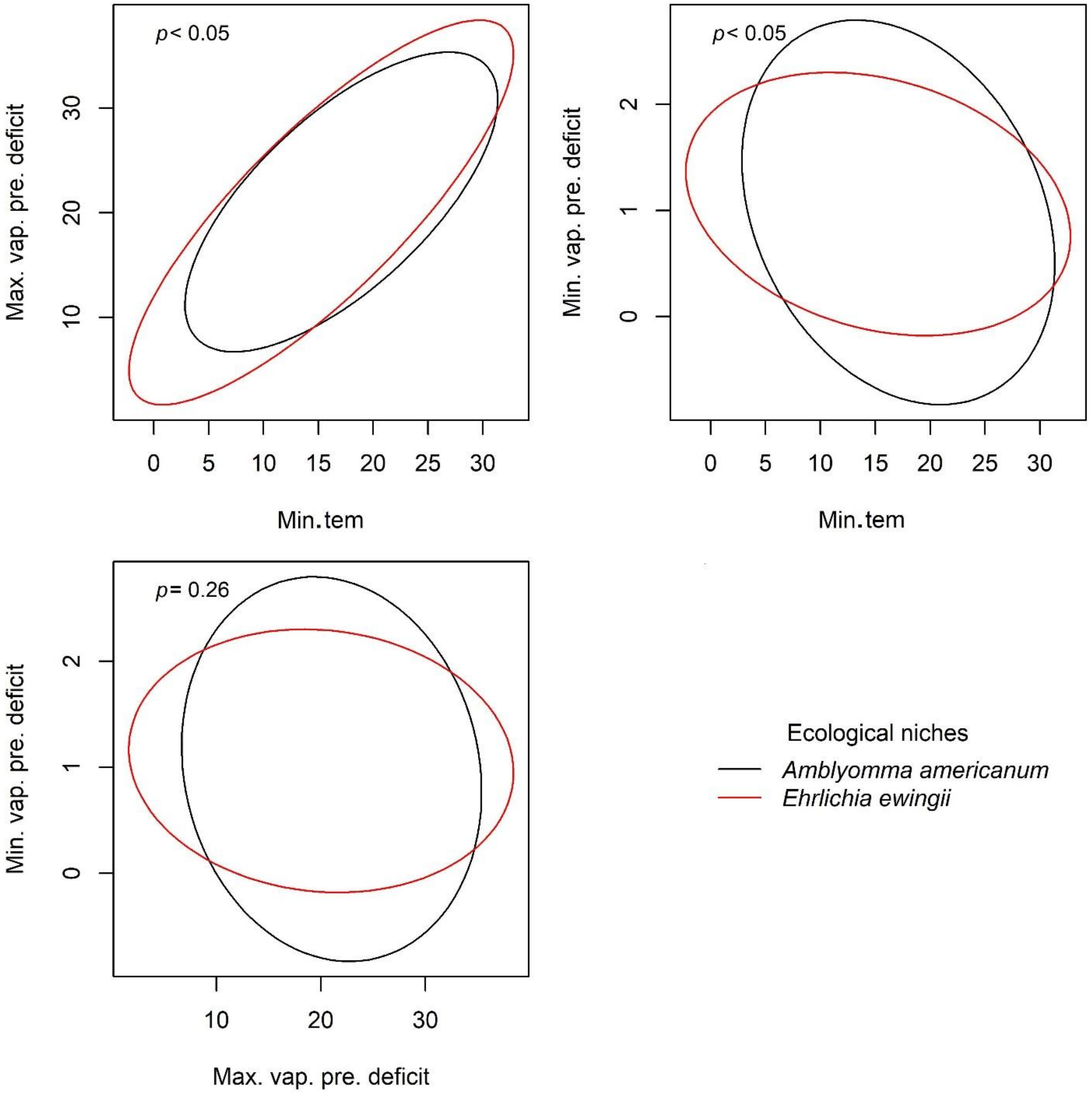

S3. PERMANOVA analysis for ecological niche of *Amblyomma americanum*, and *Ehrlichia ewingii*. Ellipses were created from occurence data, and maximum and minimum vapor pressure deficit and minimum temperature in two dimensions. Significant values are shown for each comparison.

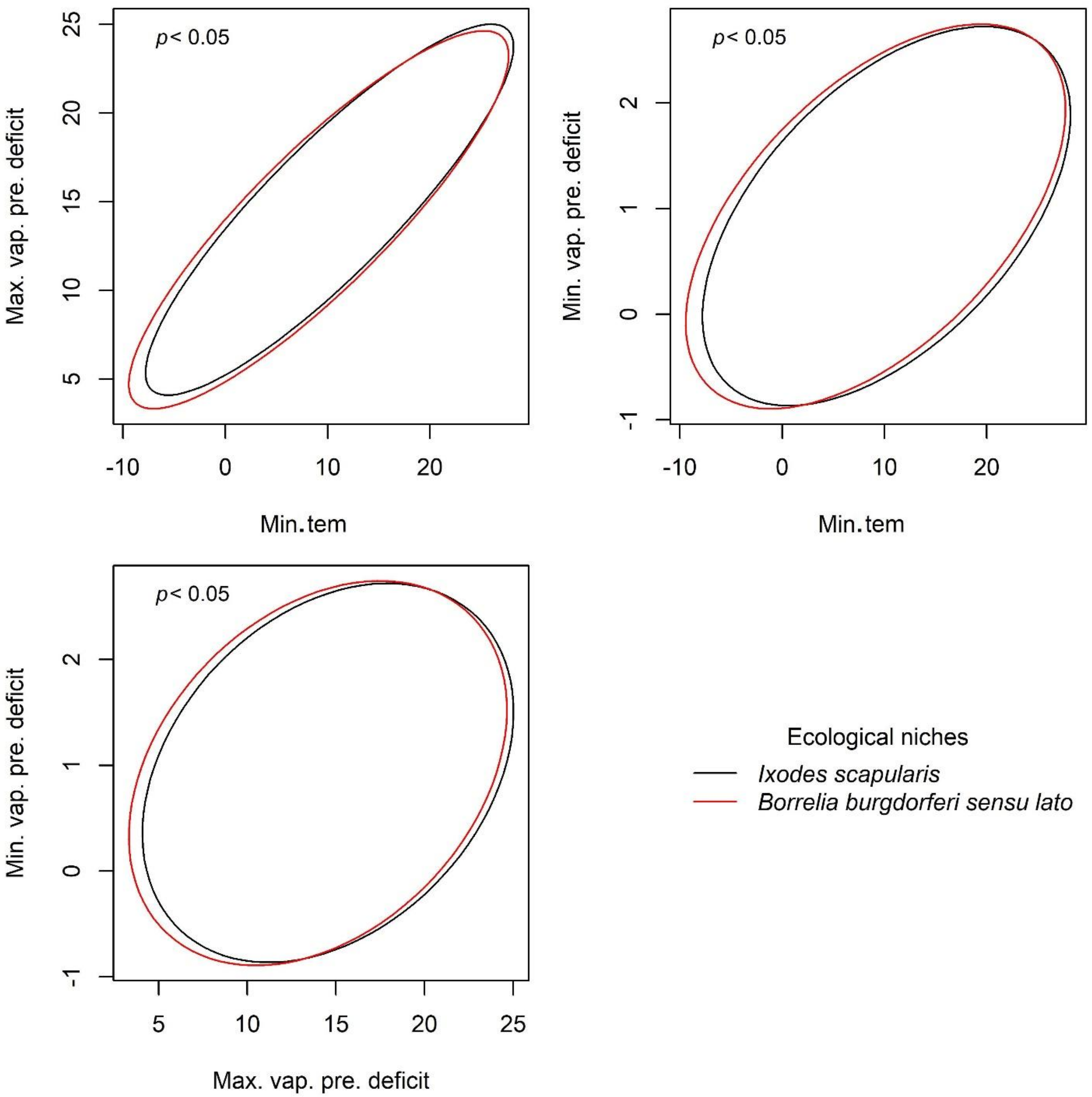

S4. PERMANOVA analysis for ecological niche of *Ixodes scapularis,* and *Borrelia burgdorferi* sensu lato. Ellipses were created from NEON data, and maximum and minimum vapor pressure deficit and minimum temperature in two dimensions. p-values are shown for each comparison.
